# VibrioBase: A Model for Next-Generation Genome and Annotation Database Development

**DOI:** 10.1155/2014/569324

**Published:** 2014-08-04

**Authors:** Siew Woh Choo, Hamed Heydari, Tze King Tan, Cheuk Chuen Siow, Ching Yew Beh, Wei Yee Wee, Naresh V. R. Mutha, Guat Jah Wong, Mia Yang Ang, Amir Hessam Yazdi

**Affiliations:** ^1^Department of Oral Biology and Biomedical Sciences, Faculty of Dentistry, University of Malaya, 50603 Kuala Lumpur, Malaysia; ^2^Genome Informatics Research Laboratory, HIR Building, University of Malaya, 50603 Kuala Lumpur, Malaysia; ^3^Department of Software Engineering, Faculty of Computer Science and Information Technology, University of Malaya, 50603 Kuala Lumpur, Malaysia; ^4^Department of Computer System & Technology, Faculty of Computer Science and Information Technology, University of Malaya, 50603 Kuala Lumpur, Malaysia

## Abstract

To facilitate the ongoing research of *Vibrio* spp., a dedicated platform for the *Vibrio* research community is needed to host the fast-growing amount of genomic data and facilitate the analysis of these data. We present VibrioBase, a useful resource platform, providing all basic features of a sequence database with the addition of unique analysis tools which could be valuable for the *Vibrio* research community. VibrioBase currently houses a total of 252 *Vibrio* genomes developed in a user-friendly manner and useful to enable the analysis of these genomic data, particularly in the field of comparative genomics. Besides general data browsing features, VibrioBase offers analysis tools such as BLAST interfaces and JBrowse genome browser. Other important features of this platform include our newly developed in-house tools, the pairwise genome comparison (PGC) tool, and pathogenomics profiling tool (PathoProT). The PGC tool is useful in the identification and comparative analysis of two genomes, whereas PathoProT is designed for comparative pathogenomics analysis of *Vibrio* strains. Both of these tools will enable researchers with little experience in bioinformatics to get meaningful information from *Vibrio* genomes with ease. We have tested the validity and suitability of these tools and features for use in the next-generation database development.

## 1. Introduction

*Vibrio* is a genus of Gram-negative bacteria possessing a curved rod shape, of which several species are pathogenic.* Vibrio cholerae* [1] is one of the well-studied pathogenic species that causes foodborne infection and the cholera disease. Cholera is a diarrheal disease [2] that can kill within hours if it remains untreated. There are about 3 to 5 million cholera cases with 100,000 to 120,000 fatalities due to cholera infections every year [3]. Other notable members of pathogenic* Vibrio *spp. include the* Vibrio parahaemolyticus *[4],* Vibrio vulnificus *[5], and* Vibrio harveyi *[6]. These microorganisms are naturally found in saltwater and they are a leading cause of seafood-borne infections such as gastroenteritis and septicemia, carried by organisms such as crabs, prawns, or oysters. Infections with the noncholera* Vibrio* are commonly associated with eating undercooked seafood [7, 8] or open wounds infection [8]. Many other* Vibrio* spp. can also cause infections in sea-living organisms and are common causes of mortality in domestic marine life.

Many new* Vibrio* genomes have been sequenced and the availability of these genome sequences from different sources has made it possible to perform genome-wide comparative analyses [9–11]. Such comparative analysis may enhance our understanding of the biology, diversity, evolution, and virulence of the* Vibrio *bacteria, which may be useful towards combating the* Vibrio* pathogens. Recently, an online genome database, MabsBase, has been developed specifically for the* Mycobacterium abscessus, *a genus of Actinobacteria [12]. However, a similar genome database is not available for* Vibrio *spp. MBGD [13], IMG [14], PATRIC [15], and SEED database [16] do provide a wide array of microbial genomes including some of the* Vibrio* strains for comparative genomics, but without much emphasis on virulence factors for comparative pathogenomics and lacking user-friendly web interfaces. PATRIC [15] does provide virulence factors information but lack functionalities to cluster/compare and visualize user-selected* Vibrio* strains based on their virulence gene profiles, which are useful for comparative pathogenomics analysis.

To facilitate the ongoing research of* Vibrio *spp., we have developed VibrioBase, a dedicated platform for the* Vibrio* research community to host the fast-growing amount of genomic data and facilitate the analysis of these data. This online platform is empowered by advanced web technologies and in-house analysis tools for the* Vibrio* research community. The comprehensive set of genomic datasets in VibrioBase will facilitate analyses in comparative genomics and pathogenomics among different* Vibrio *strains or species. Here, we describe the overview and some key features of VibrioBase.

## 2. Database Content and Organization

VibrioBase currently houses a total of 252 genome sequences from 31* Vibrio* spp. obtained from the NCBI (Supplementary Table 1 in Supplementary Material available online at http://dx.doi.org/10.1155/2014/569324). All the genome sequences in VibrioBase were annotated using the rapid annotation subsystem technology (RAST) pipeline [19]. The RAST pipeline is a fully automated annotation engine and helps in recognizing protein encoding genes, rRNAs, and tRNAs and subsequently ascribes functions to the genes. The protein assignments in the RAST pipeline are based on the functional properties within the subsystems (see the subsystems approach to genome annotation and its use in the project to annotate 1000 genomes) and FIGfams [20] maintained in the SEED system. The annotations include the functional prediction, protein translation of contigs, RNA and CDS classification, subsystem function prediction, and the start and stop positions of sequences. The PSORTb standalone version 3.0 [21] was used to predict the subcellular localization of each putative protein in the VibrioBase datasets. Protein subcellular localization describes the spatial arrangement of proteins within cells, thereby providing important functional information of proteins. The objective of this database is to provide resources for whole-genome annotations and state-of-the-art tools to support the expanding* Vibrio* research community and to collectively gather information on the strains of* Vibrio* spp. into a single database. With this annotation information, users are a step closer to the understanding of* Vibrio *spp. Information of all the strains in this database is linked to the NCBI database. For instance, by following the ORF ID, users will be taken to the NCBI database for further information on that specific region of each particular ORF. We present here some of the key features in our database which would be quintessential for the next-generation genome and annotation database development.

## 3. Real-Time Data Searching Feature

With the advances in next-generation sequencing technologies and bioinformatics tools, more* Vibrio* genomes and annotations will be progressively included in VibrioBase. Our goal is to enable users to browse rapidly and seamlessly along the huge genomic data. VibrioBase can be queried to obtain annotated features in different ways. We facilitated this by implementing a powerful real-time AJAX-based search function in this database. The AJAX is not a new technology but a combination of different existing technologies such as HTML, CSS, DOM, XML, and JavaScript. This allows our database to have a wider variety of controls and functions. It reduces the load on server which allows heavy analyses load to be processed simultaneously. Users can perform quick browsing of the database using keywords in a rapid, real-time manner which is a very important feature for browsing through database with massive datasets. The interactive JBrowse [22] genome browser incorporated in VibrioBase which uses the AJAX-based, server and client technology support fast and smooth animated genome navigation over the web. As we anticipate that many more Vibrio genomes are to be sequenced and annotated, the use of user friendly genome browser, the JBrowse, has become essential. Unlike other genome browsers such as the GBrowse, which is implemented using the Common Gateway Interface (CGI) protocol occupying much more resources and delaying in response for the out coming result, the server-client architecture of JBrowse minimizes server overhead problems as genomes are not rendered into images on the web server. This provides the users to have rapid genome browsing information without causing server overload when multiple users process simultaneously. JBrowse provides efficient panning and zooming of a genomic region in the genome via embedded navigation buttons, thus avoiding discontinuous transitions. Furthermore, JBrowse allows each track such as DNA, RNA, and CDS tracks to be turned on or off on clicking it. It allows users to hide unwanted information for better user experience.

## 4. Pairwise Genome Comparison (PGC) Tool: Information Aesthetic for Comparative Genomics

**Figure 1:**
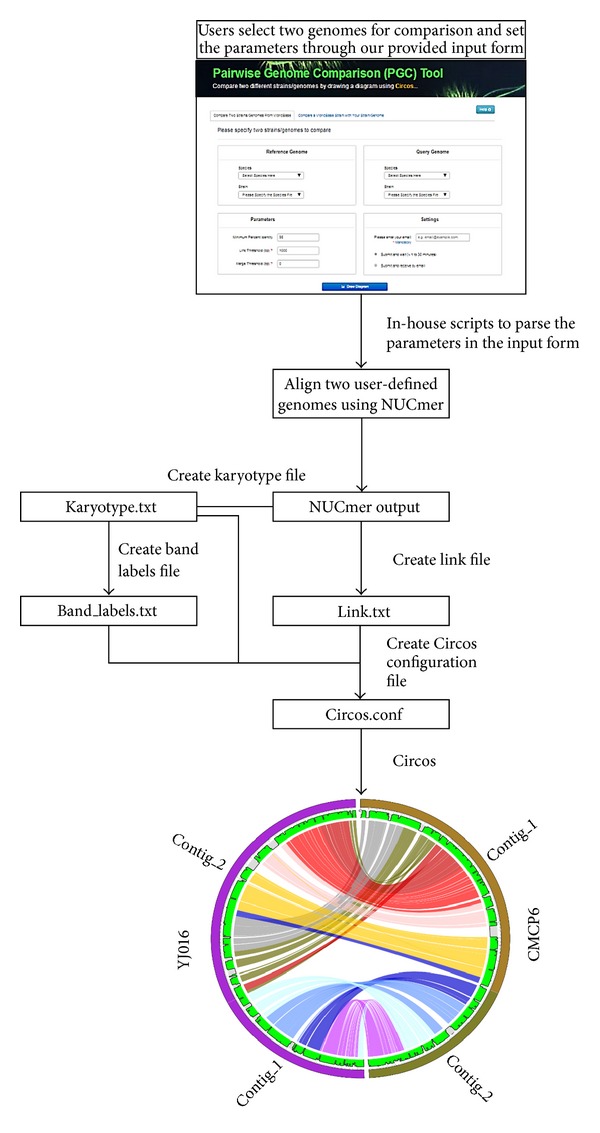
A workflow of the PGC tool. Through an input web interface on VibrioBase, users can choose two genomes of interest (reference versus query genomes) in VibrioBase and parameters for comparison. Three available useful parameters/thresholds which are the minimum percent identity, merge threshold, and link threshold. The minimum percent identity parameter will display aligned genomic regions (represented by colored links) once the sequence identity is higher than the user-defined cut-off. Similar to the merge threshold, this threshold will merge two links if the distance between the two regions is lower than the user-defined cut-off. The link threshold ignores links in the diagram if their widths are lower than the user-defined cut-off. Once the job is submitted to our server, PGC pipeline parses this information and starts the genome alignment with NUCmer. A series of in-house Perl and Python scripts are used to parse the NUCmer output and generate different text files: (1) Karyotype.txt stores information about the contigs and their colors used in Circos; (2) Links.txt stores information about the aligned genomic regions; (3) Band_labels.txt keeps the names of each contigs/chromosome. The Circos.conf configuration file will be created using the information in the above four files, which is needed for displaying the two aligned genomes with Circos.

The pairwise genome comparison (PGC) tool is a newly designed in-house comparative analysis tool integrated to allow users to compare two different* Vibrio* genomes (Figure 1). Two main software components used in the automated pipeline were NUCmer in the MUMmer package [23] and Circos [24]. This visualization tool is useful in the identification and comparative analysis of two genomes which is reported in a circular ideogram layout, depicting the structural variations and positional relationships between two genomic intervals. The input form of PGC has three parameters which are the minimum percent identity, merge threshold, and link threshold. By default, the Circos in VibrioBase is set to 95% minimum percentage identity and 1,000 bp link threshold; users may change the parameter freely to get different comparative results.

**Figure 2:**
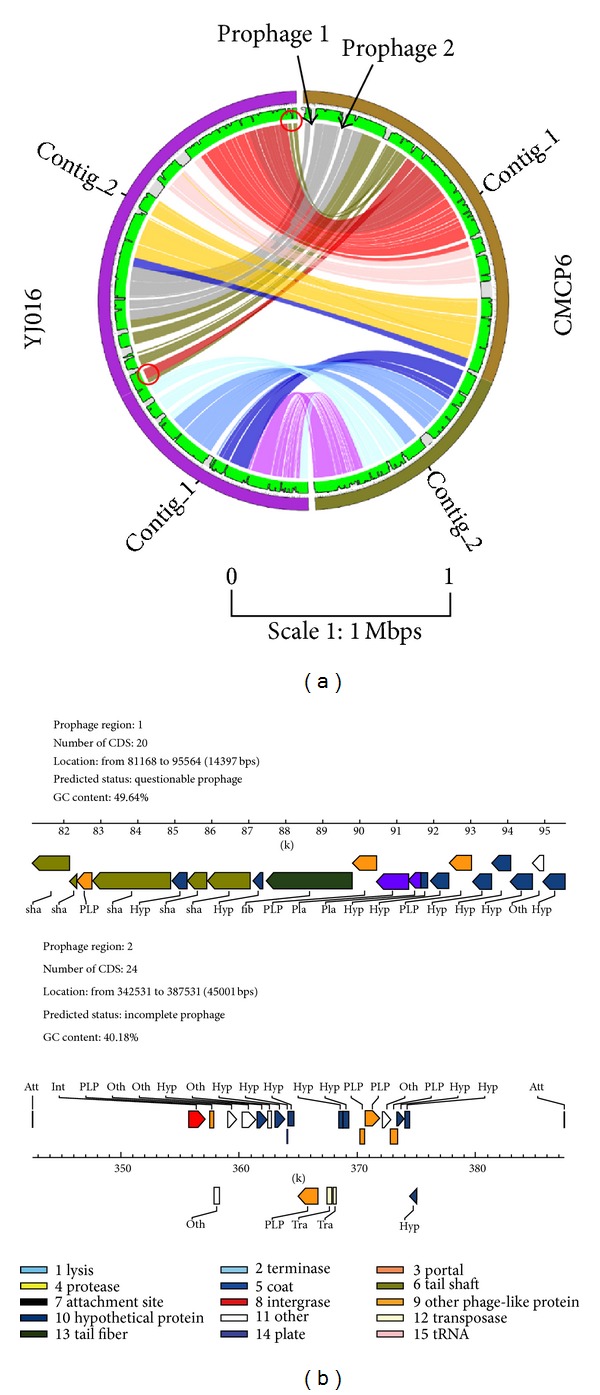
Example of a pairwise genome alignment between* V. vulnificus* YJ016 and* V. vulnificus* CMCP6. (a) Circos plot reveals differences between the two genomes. Red circle indicates the translocations possibly occur. Black arrowheads indicate phage insertions. (b) The structure of the two putative prophages. (Scale of the Circos 1 : 1 Mbps).

As an example, we compared the genomes of two closely related* Vibrio* species:* V. vulnificus* CMCP6 and* V. vulnificus* YJ016. The two genomes are very similar or conserved in general. Interestingly, Circos plot has shown that there are two putative genomic translocations in the genome of * V. vulnificus* YJ016 and indels in the two genomes (Figure 2). Further analysis on the sequences of these regions revealed that the two regions in the genome of* V. vulnificus* CMCP6 are associated with putative prophages as predicted by PHAST [25], suggesting that these prophages might be inserted into* V. vulnificus* CMCP6. The insertion of prophages might be due to previous lysogenic bacteriophage infection and this probably confers pathogenicity to the bacterial host and changes its diversity. Here, we have demonstrated that PGC can be used to study the genetic differences between two genomes.

Although a similar tool named Circoletto [26] is available, there are many differences between this tool and our newly developed PGC. For instance, Circoletto aligns sequences using BLAST (local alignment), but PGC uses the NUCmer (global alignment) package in MUMmer 3.0 [27], which is advantageous for large-scale and rapid genome alignment. Moreover, PGC also allows users to adjust settings such as minimum percent identity, merge threshold, and link threshold through the provided web interface.

## 5. Pathogenomics Profiling Tool (PathoProT)

**Figure 3:**
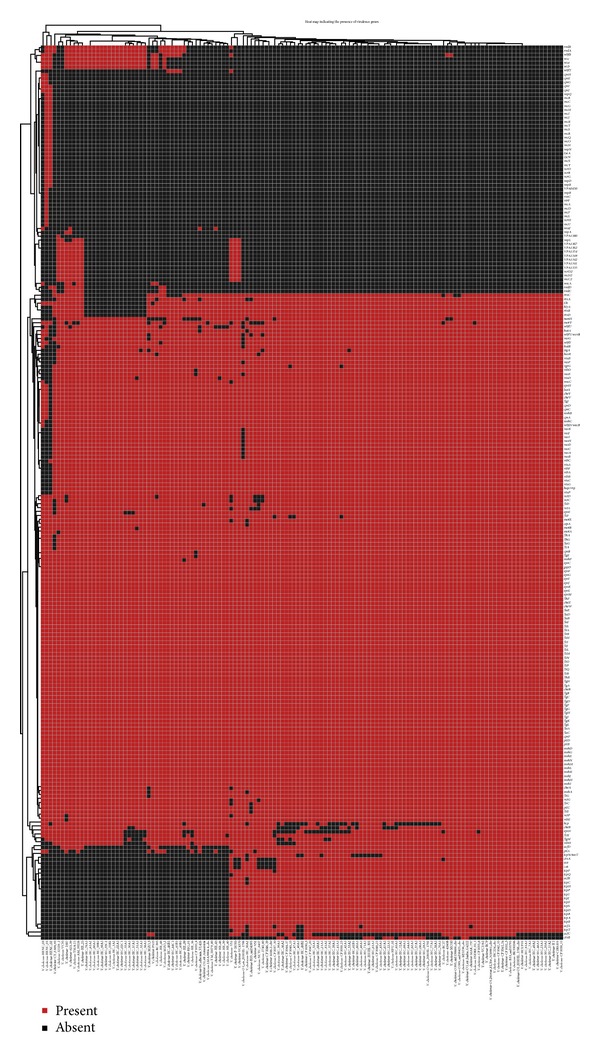
The profiling of virulence genes of 134* V. cholerae* strains using PathoProT. The heat map was generated by using the thresholds of 50% completeness and 50% identity. It gave an overview of the clustered strains having closely related sets of virulence genes, sorted according to the level of similarities across the strains and genes. The flagella formation gene clusters (*flgB-flgC-flgD-flgF-flgG-flgH-flgI-flgK-flgL*) are the largest conserved virulence gene group among the* V. cholera *strains. The* flg* gene cluster works with other virulence gene clusters such as* fla* gene cluster (*flaA-flaB-flaC-flaD-flaE*) [17] and * fli* gene cluster (*fliH-fliJ-fliL-fliM-fliN-flnO-fliP-fliQ-fliR-fliS*) to form the flagella organelles on the bacteria [18], which is one of the common characteristics among all cholerae strains.

We developed a unique pathogenomics profiling tool (PathoProT) that could identify virulence genes and perform a comparative pathogenomics analysis. PathoProT predicts the virulence genes based on a sequence homology search of the putative protein sequences in each strain against the virulence factors database (VFDB) [28], depending on the user-defined cut-offs for protein identity and completeness. This tool clusters (agglomerative hierarchical cluster analysis) the predicted virulence genes across different strains using their virulence gene profiles. A user can visualize them in the form of heat map with dendrograms. Furthermore, it allows users to examine the similarities and differences of the virulence gene profiles between different groups of strains, for instance, nonpathogenic versus pathogenic strains. We compared the virulence gene profiles of all 134 strains of the highly pathogenic* V. cholera* in VibrioBase using PathoProT (Figure 3). Our analysis showed that all strains shared at least 58 conserved virulence genes, amongst which is the exopolysaccharide genes group (*epsC-epsF-epsG-epsI-epsJ-epsK-epsL-epsM*) [29–31] responsible for carrying out different virulence functions such as protection of bacteria from phagocytosis, phage invasion, and protection of cell against water absorption [32]. Besides showing the conserved virulence genes, the heat map is also able to show strain-specific or group-specific virulence genes.

**Figure 4:**
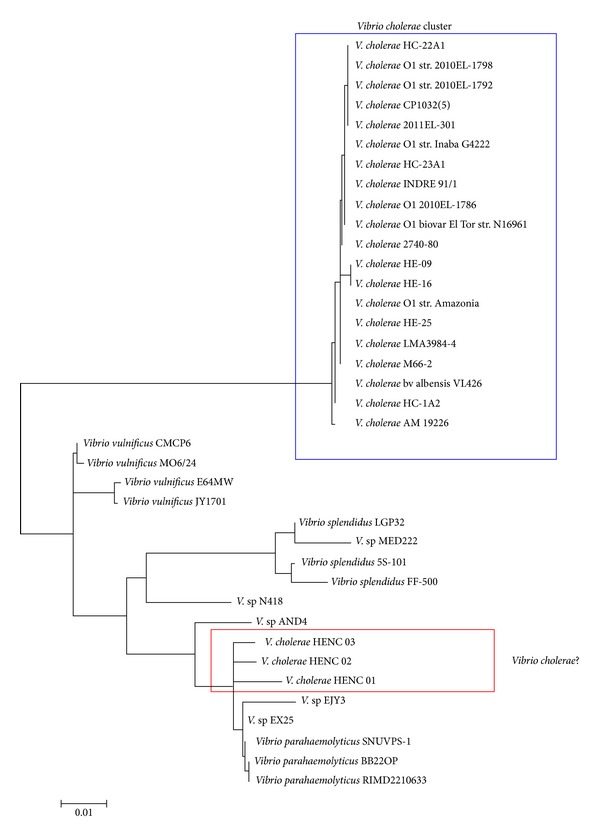
A 16S-based phylogenetic tree.* V. cholera *HENC 01,* V. cholera *HENC 02, and* V. cholera *HENC 03 are clustered into a group (red box), rather than with other* V. cholera *strains (blue box). The three strains are closely related to* V. parahaemolyticus *species. These results suggest that the three strains might be inappropriately classified into* V. cholera*.

Interestingly, three strains (*V. cholerae *HENC 01,* V. cholerae* HENC 02, and* V. cholera* HENC 03) have very different virulence profiles as compared to other* V. cholerae *strains (Figure 4). One of the possible explanations is that these genes were not completely sequenced because the genomes of these strains are not complete. However, this may be unlikely because we observed that many virulence genes are also absent in the three genomes. Another possibility is that these strains were inappropriately classified as* V. cholerae*. To investigate the second possibility, we constructed a phylogenetic tree based on the 16S rRNA genes from different* Vibrio* spp. with MEGA4 using the maximum likelihood methods [27]. We found that these three strains are clustered into a group (Figure 4), but not with other* V. cholerae* strains, supporting our view that these three strains are not of* V. cholera*. It should be noted that, at the end of our analysis, these strains were recently renamed in the NCBI database to uncategorized* Vibrio* spp. Taken together, we have shown that PathoProT can be a useful tool for comparative pathogenomics analysis of the* Vibrio* strains or species, which may enhance our understanding of their virulence, evolution, and classification.

## 6. Implementation and Future Development

VibrioBase is a HTML5 web application developed in PHP language using CodeIgniter and Twitter Bootstrap as back-end and front-end frameworks, respectively. MySQL database is used for storing information and the webserver is configured using Open Panel. To achieve higher level of performance, a job management and scheduler were developed to manage and monitor jobs, which include Circos plots and BLAST search. AJAX calls were emerged in different components of the application to provide a better user experience.

VibrioBase will be updated from time to time as more genome annotations and genomic sequences of* Vibrio *become available. To accelerate the development of this database for the use of the scientific community, we encourage other research groups to contact us if they would like to share annotations and related datasets with us. Suggestions on improving VibrioBase and requests for additional functions are also welcome.

## 7. Conclusion

Although there are many existing valuable genomic data and analysis tools from different sources, it is important to pool these resources together in order to allow rapid searching and browsing and also to facilitate the analysis of these data. We believe that a specialized resource such as the VibrioBase that is empowered with different analysis tools will prove to be invaluable to the* Vibrio* researchers both in basic science and clinical research areas.

## Supplementary Material

Supplementary Table 1 shows the list of Vibrio species and strains available in VibrioBase. VibrioBase currently hosts a total of 252 genome sequences or strains of Vibrio species retrieved from the NCBI database. There are 24 complete genomes and 228 draft genomes.
