# Supplementary material for: VibrioBase: A Model for Next-Generation Genome and Annotation Database Development
Source: ScientificWorldJournal. 2014 Aug 4;2014:569324. doi: 10.1155/2014/569324 (PMC4138799; doi:10.1155/2014/569324)
Supplement: Supplementary file 1 — Supplementary Table 1 shows the list of Vibrio species and strains available in VibrioBase. VibrioBase currently hosts a total of 252 genome sequences or strains of Vibrio species retrieved from the NCBI database. There are 24 complete genomes and 228 draft genomes. [file 569324.f1.pdf]

Supplementary Data

Supplementary Table 1. List of *Vibrio* species in VibrioBase.

#	Species	Number of Draft Genomes	Number of Complete Genomes
1	***V. alginolyticus***	3	0
2	***V. anguillarum***	2	1
3	***V. brasiliensis***	1	0
4	***V. breoganii***	3	0
5	***V. campbellii***	3	0
6	***V. caribbenthicus***	1	0
7	***V. cholerae***	124	10
8	***V. coralliilyticus***	2	0
9	***V. crassostreae***	5	0
10	***V. cyclitrophicus***	20	0
11	***V. fischeri***	0	3
12	***V. furnissii***	1	1
13	***V. harveyi***	4	1
14	***V. ichthyoenteri***	1	0
15	***V. kanaloae***	1	0
16	***V. metschnikovii***	1	0
17	***V. mimicus***	6	0
18	***V. nigripulchritudo***	1	0
19	***V. ordalii***	6	0
20	***V. orientalis***	2	0
21	***V. parahaemolyticus***	7	2
22	***V. rotiferianus***	1	0
23	***V. rumoiensis***	1	0
24	***V. scophthalmi***	1	0
25	***V. shilonii***	1	0
26	***V. sinaloensis***	1	0
27	***V. sp***	12	1
28	***V. splendidus***	5	2
29	***V. tasmaniensis***	5	0
30	***V. tubiashii***	2	0
31	***V. vulnificus***	5	3
